# Bioavailability of Macroelements from Synbiotic Sheep’s Milk Ice Cream

**DOI:** 10.3390/nu15143230

**Published:** 2023-07-20

**Authors:** Magdalena Kowalczyk, Agata Znamirowska-Piotrowska, Magdalena Buniowska-Olejnik, Grzegorz Zaguła, Małgorzata Pawlos

**Affiliations:** 1 Department of Dairy Technology, Institute of Food Technology and Nutrition, College of Natural Sciences, University of Rzeszow, Ćwiklińskiej 2D, 35-601 Rzeszów, Poland; mbuniowska@ur.edu.pl (M.B.-O.); mpawlos@ur.edu.pl (M.P.); 2 Department of Bioenergetics, Food Analysis and Microbiology, Institute of Food and Nutrition Technology, College of Natural Science, University of Rzeszow, Ćwiklińskiej 2D, 35-601 Rzeszów, Poland; gzagula@ur.edu.pl

**Keywords:** ice cream, sheep milk, probiotic, prebiotic, macroelements, bioavailability, in vitro

## Abstract

To determine the potential bioavailability of macroelements (Ca, Mg, P, K), probiotic ice cream samples (*Lactaseibacillus paracasei* L-26, *Lactobacillus casei* 431, *Lactobacillus acidophilus* LA-5, *Lactaseibacillus rhamnosus* and *Bifidobacterium animalis* ssp. *lactis* BB-12) from sheep’s milk with inulin, apple fiber and inulin, or apple fiber and control samples were submitted to in vitro digestion in the mouth, stomach and small intestine. The bioavailability of calcium in the ice cream samples ranged from 40.63% to 54.40%, whereas that of magnesium was 55.64% to 44.42%. The highest bioavailability of calcium and magnesium was shown for the control samples. However, adding 4% inulin reduced the bioavailability of calcium by about 3–5% and magnesium only by about 5–6%. Adding 4% apple fiber reduced the bioavailability of calcium by as much as 6–12% and magnesium by 7–8%. The highest bioavailability of calcium was determined in ice cream with *L. paracasei*, and the highest bioavailability of magnesium was determined in ice cream with *L. casei*. The bioavailability of phosphorus in ice cream ranged from 47.82% to 50.94%. The highest bioavailability of phosphorus (>50%) was in sheep ice cream fermented by *B. animalis*. In the control ice cream, the bioavailability of potassium was about 60%. In ice cream with inulin, the bioavailability of potassium was lower by 3–4%, and in ice cream with apple fiber, the bioavailability of potassium was lower by up to 6–9%. The bioavailability of potassium was significantly influenced only by the addition of dietary fiber. The results of the study confirmed the beneficial effect of bacteria on the bioavailability of Ca, Mg and P.

## 1. Introduction

Sheep’s milk is a valuable source of minerals. Macronutrients occur in sheep’s milk in substantially higher concentrations than in cow’s or goat’s milk (calcium 193 mg 100 g^−1^, magnesium 18 mg 100 g^−1^, phosphorus 158 mg 100 g^−1^, sodium 44 mg 100 g^−1^ and potassium 136 mg 100 g^−1^) [1,2].

In milk, micelles are bound by calcium phosphate and small amounts of magnesium and citrate [3]. Micelles are characterized by different sizes in the milk of various animal species [4,5]. Camel milk features micelles with the largest diameter (380 nm), while goat milk is 260 nm and sheep milk is 180 nm, which may also affect the variations in the bioavailability of macronutrients. Another characteristic of sheep’s milk is the richness of vitamin D, which promotes calcium absorption and contributes to the proper development of the skeletal system [6].

The mineral composition may differ from the content in dairy products. It was found that such differences may be the consequence of the processing factors applied, including heat treatment, homogenization or pasteurization, fermentation, type of starter culture and fermentation time, as well as storage conditions and time [7,8,9]. These factors generally lead to structural changes, which may affect digestion, the kinetics of mineral delivery and, consequently, the bioavailability of these components [10,11].

Minerals are mainly absorbed in the small intestine. The bioavailability of minerals strongly depends on a human’s age and health status, the type of product consumed, the form of the compound, the interaction between minerals contained in the product, technological processing and pH. The bioavailability of minerals is significantly influenced by probiotic bacteria, gastrointestinal microflora and prebiotics [12].

Enzymes in the small intestine do not digest prebiotics such as inulin and apple fiber, and these prebiotics stimulate the growth and activity of probiotic bacteria by undergoing fermentation [13,14], play essential roles in the prevention and treatment of disease, lower sugar and cholesterol levels and contain minerals. Numerous studies indicate that inulin and oligosaccharides, especially fructooligosaccharides (FOSs), may increase the absorption of minerals, including calcium and magnesium [15,16,17,18].

Probiotic bacteria colonizing the intestines are believed to contribute to the bioavailability of calcium and magnesium [19,20,21,22]. Bioavailability is defined as the intake of a nutrient in food that passes through the gastrointestinal tract, is absorbed, reaches tissues and is used in physiological functions of the body or stored. Therefore, bioavailability studies are essential to better assess the mineral content provided by foods. An effective way to determine the bioavailability of nutrients is the use of in vitro digestion models.

As a popular dessert consumed worldwide, dairy ice cream can be an excellent carrier of minerals. Adding probiotic bacteria, inulin and apple fiber could potentially help improve the bioavailability of macronutrients, a crucial issue. There is no information on the bioavailability of Ca, K, Mg and P from sheep’s milk ice cream manufactured from mixes fermented by probiotic strains. The differences in milk composition (macro- and microscale) of different species mean that further research is needed to determine the most appropriate types of milk and milk products to improve the bioavailability of micro- and macronutrients [22,23,24]. Therefore, to determine the potential bioavailability of macronutrients (Ca, Mg, P, K), probiotic ice cream samples (*Lactaseibacillus paracasei* L-26, *Lactobacillus casei* 431, *Lactobacillus acidophilus* LA-5, *Lactaseibacillus rhamnosus* and *Bifidobacterium animalis* ssp. *lactis* BB-12) from sheep milk with inulin, apple fiber and inulin, or apple fiber and control samples were subjected to in vitro enzymatic digestion.

## 2. Materials and Methods

### 2.1. Materials

Digestive enzymes: heat-stable α-amylase (TDF-100A, 24 975 U ML^−1^), mucin from the porcine stomach (type II), pepsin from the porcine gastric mucosa (250 U mg^−1^ solid), porcine bile extract and pancreatin from the porcine pancreas (8 × USP specifications) were provided by Sigma-Aldrich (St. Louis, MO, USA).

Reagents: di-sodium hydrogen phosphate anhydrous pure p.a. ≥99.0% (Na_2_HPO_4_; 141.96 g mol^−1^), di-potassium hydrogen phosphate (K_2_HPO_4_; 174.18 g mol^−1^), sodium chloride pure p.a. ≥99.9% (NaCl; 58.44 g mol^−1^), hydrochloric acid (12 M HCl) and sodium hydroxide (1 M NaOH) were supplied by Chempur (Piekary Śląskie, Poland). All of the reagents used were of analytical reagent grade. Nitric acid (HNO_3_) was provided by Sigma Aldrich (St. Louis, MO, USA). EDTA disodium salt (EDTA Na_2_ 0.01 M), sodium bicarbonate (NaHCO_3_; 2%), sodium bicarbonate (NaHCO_3_ 0.5 M) and sodium dodecyl sulfate (0.1%) were also purchased from Sigma-Aldrich (St. Louis, MO, USA). MRS agar and peptone media were purchased from Biocorp (Warszawa, Poland). The cellulose membrane for dialyzing (avg. flat width 25 mm, molecular weight cut-off = 14,000) was purchased from Sigma-Aldrich (St. Louis, MO, USA).

Raw sheep’s milk for the production of ice cream mixtures was obtained from a farm in Wyżne, Podkarpacie, Poland (5.34 ± 0.2% protein, 6.20 ± 0.3% fat, 5.01 ± 0.12% lactose, pH 6.8 ± 0.12). Inulin (carbohydrates 97 g 100 g^−1^, including sugars 7 g 100 g^−1^, fiber 90 g 100 g^−1^, fat 0 g 100 g^−1^ and protein 0 g 100 g^−1^; Orafti HP, Oreye, Belgium), 100% micronized apple fiber (carbohydrates 87 g 100 g^−1^, including sugars 27 g 100 g^−1^, fiber 51 g 100 g^−1^, fat 3.3 g 100 g^−1^ and protein 5.1 g 100 g^−1^; Aura Herbals Jarosław Paweł, Sopot, Poland) and white sugar (Cukier Polski, Toruń, Poland) were used as additives.

Probiotic bacteria used for fermentation: *Lactaseibacillus paracasei* L-26, *Lactobacillus casei* 431, *Lactobacillus acidophilus* LA-5, *Lactaseibacillus rhamnosus* and *Bifidobacterium animalis* ssp. *lactis* BB-12 were purchased from Chr. Hansen (Hoersholm, Denmark). Freeze-dried commercial starter cultures used in the dairy industry are not genetically modified.

### 2.2. Experimental Design and Ice Cream Manufacture

**Figure 1:**
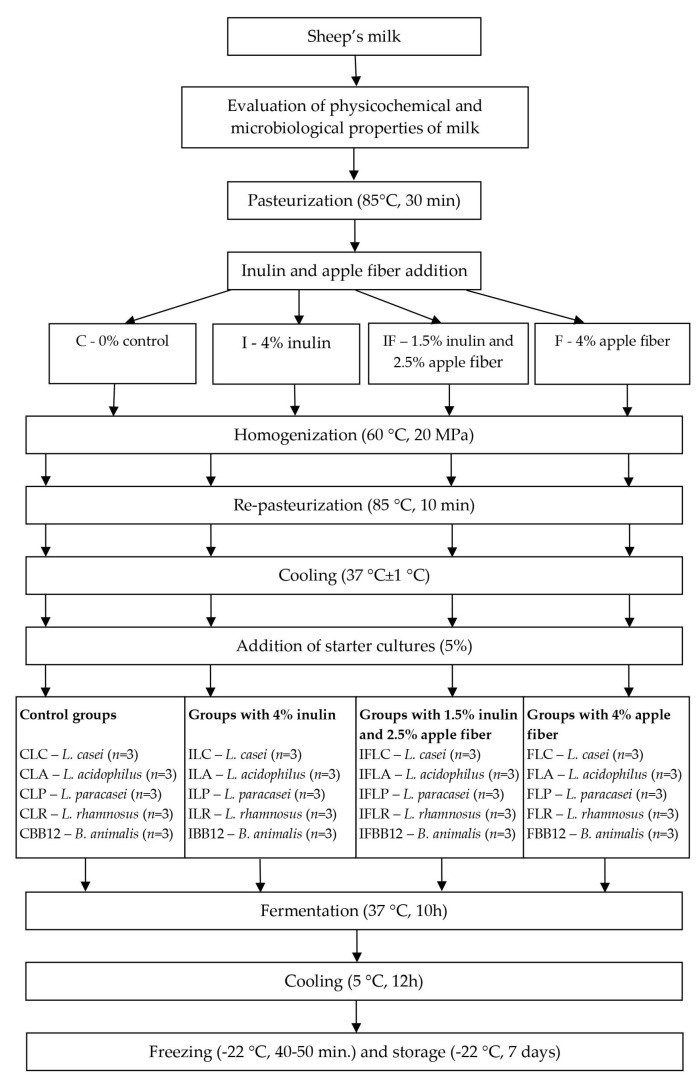
Manufacture of ice cream from sheep’s milk.

The process of producing ice cream from sheep’s milk according to the method described by Kowalczyk et al. [25] is included in Figure 1. For each probiotic strain, four batches of mixtures with additives were produced: C—sheep milk (89%) with sugar (11%); I—sheep milk (85%), sugar (11%) and inulin (4%); IF—sheep milk (85%), sugar (11%), inulin (1.5%) and apple fiber (2.5%); F—sheep milk (85%), sugar (11%) and apple fiber (4%). Each batch was homogenized (CAT UNIDRIVE X 1000 D, Ballrechten-Dottingen, Germany) and pasteurized (85 °C, 1 min). After the heat treatment, mixes were cooled to 37 °C, inoculated with one of five previously revived monocultures of probiotic bacteria at 5% (*w*/*w*) and fermented for ten hours in an incubator (cooled incubator ILW 115, POL-EKO-Aparatura, Wodzisław Śląski, Poland), and then cooled to 5 °C and conditioned for 12 h. The ice cream mixtures were frozen in a DeLux 48,816 freezer (UNOLD AG, Hockeheim, Germany) for 40–50 min at −22 °C [25]. Next, the ice cream samples were stored in 100 mL plastic cups at −22 °C for seven days until simulated in vitro digestion was performed. The experiment was repeated three times, and all analyses were performed in three replicates each time.

### 2.3. In Vitro Digestion Process

The procedure of simulated digestion in the gastrointestinal tract was carried out according to the methods presented by Buniowska et al. [26] and Camelo-Silva et al. [27] with some modifications. All ice cream samples were digested after seven days of storage at −22 °C. The simulated digestive system included the oral stage stomach and small intestine. The pH, enzymes and time were adjusted for each in vitro digestion step.

To simulate the oral stage, 50 mL of sample and 5 mL of saliva enzyme solution (2.38 g Na_2_HPO_4_, 0.19 g K_2_HPO_4_, 8 g NaCl, 100 mg L^−^^1^ mucin and 150 mg L^−^^1^ α-amylase with enzyme activity of 200 U L^−^^1^, dissolved in 1 L distilled water) were transferred to a dark glass bottle. Using HCl (12 mol L^−^^1^) or NaOH (1 mol L^−^^1^) buffers, the mixture was adjusted to pH 6.75 ± 0.20 and then incubated in a shaker at 37 *°C* and 90 rpm for 10 min. In particular, 10 min is an extended time relative to the average time food is in the mouth during chewing, but this value ensured repeatability between samples. After the oral stage, the simulation of the gastric phase was initiated. In this stage, 13.08 mg of pepsin was added to the sample mixed with saliva, and the pH value was reduced to 2.0 ± 0.20 by adding HCl (12 mol L^−^^1^). The sample was placed in a shaker for 2 h at 37 °C and 90 RPM.

To induce simulated intestinal digestion, the contents from the oral and gastric stages were mixed with 5 mL of pancreatin (4 g L^−1^) and bile salt (25 g L^−1^), changing the pH value to 7.00 ± 0.20 (12 M HCl M or 1 M NaOH). The previously prepared cellulose dialysis membranes (25 mm wide and 30 cm long) were conditioned (0.01 M EDTA Na_2_, 2% NaHCO_3_ and 0.1% sodium dodecyl sulfate) at a temperature of 100 °C for 10 min and rinsed five times with deionized water, filled with 25 mL of NaHCO_3_ (0.5 M) and placed in the sample until a pH value of 7.00 ± 0.20 was reached. During this process, the pH gradually adjusted, simulating intestinal conditions. The incubation was continued for another 2 h (37 °C/90 RPM). To complete the digestion process, the sample was placed in an ice bath for 10 min. Macroelements that diffuse into the semi-permeable dialysis membrane are potentially absorbed into the bloodstream, known as dialysate. Using a dialysis membrane eliminates the problems that occur during the dialysis of soluble and insoluble compounds [28]. The solution remaining inside the dialysis membrane is the sample component potentially absorbed into the bloodstream.

Bioavailability (%) refers to the percentage of the mineral compound tested that remains in the dialyzed intestinal fraction relative to the original undigested sample (before digestion), according to the following equation:

$$\mathrm{Bioavailability}=100\;\times \;\frac{\;Dialysed\;fraction\;}{Before\;digestion}$$

After seven days, samples of the product and each stage and dialysate were frozen and stored at −45 °C until further analysis (about 12–14 days).

### 2.4. ICP-OES Analysis

Macroelements in ice cream samples were measured before digestion and after each stage of in vitro digestion using optical emission spectrometry with horizontal plasma and detection capabilities both along and across the plasma flame (axial and radial) (ICP-OES) using a Thermo iCAP Dual 6500 spectrometer (Thermo Fisher Scientific Inc., Bridgewater, MA, USA) according to the method of Znamirowska et al. [29].

The instrument was calibrated with certified standards (Merck, Darmstadt, Germany) covering macronutrient concentrations (Ca, Mg, P, K) of 10,000 ppm. The method was validated using certified reference material (NIST-1515). Results were expressed in mg 100 g^−1^ dry matter.

Before the determination, the test samples were subjected to out-of-pressure mineralization in 65% HNO_3_ using a Milestone Ethos Ultra-wave-One mineralizer (Milestone SRL, Sorisole, Italy). The required amount of sample was placed in Teflon vessels, after which it was topped up with 8 mL of nitric acid and sealed tightly. The rotary mineralizer was topped up with a blank sample (8 mL of HNO_3_) during the mineralization process for all samples. The analyzed samples were mineralized for one hour using the temperature build-up algorithm, according to the procedure for this type of biological samples, not exceeding 200 °C. After mineralization, the samples were brought to room temperature, and the contents were poured into Falcon tubes (50 mL) topped up with demineralized water.

The detection level for each element was not less than 0.1 mg kg ^−1^ (assuming detection of the measuring apparatus at 10 ppb).

### 2.5. Statistical Analysis

Statistical analysis was performed using Statistica v. 13.1 (StatSoft, Tulsa, OK, USA). The mean and standard deviation were statistically calculated from the obtained results. A one- and two-factor ANOVA analysis of variance was performed. The significance of differences between the means was determined by Tukey’s test (*p* ≤ 0.05).

## 3. Results and Discussion

### 3.1. Calcium and Phosphorus

Dietary calcium intake does not meet the recommendations in many parts of the world. Dairy products provide a high level of calcium per serving, which becomes bioavailable under conditions in the gastrointestinal tract [30,31]. Crucial in sheep’s milk ice cream is the presence of casein micelles, which are protein colloids containing ~70% of total calcium and ~50% of total inorganic phosphate [32]. Calcium phosphate is enclosed in casein micelles as small nanoclusters, typically 4–5 nm in diameter. Therefore, casein micelles, which contain several hundred nanoclusters of calcium phosphate and tens of thousands of casein molecules, could be considered a protein-based carrier of calcium phosphate [33,34]. To provide adequate transport, some of the calcium from ice cream is complexed with citrate, which is present in milk at a level of 0.20 mg 100 g^−1^ [35]. The proper ratio of calcium to phosphorus in the diet is also essential for adequate phosphate–calcium metabolism. For adults, a beneficial ratio is 1:1 or 1.5:1 [36,37], indicating that in the example of the sheep’s milk ice cream studied, the amount of each macroelement is highly favorable because it varies from 1.28:1 to 1.32:1 (Table 1, Table 2, Table 3, Table 4 and Table 5).

The calcium and phosphorus contents of raw materials and probiotic sheep’s milk ice cream are shown in Table 1, Table 2, Table 3, Table 4, Table 5 and Table 6.

In all control ice cream groups (CLC, CLA, CBB12, CLP, CLR), the calcium content ranged from 199.47 mg 100 g^−1^ to 202.45 mg 100 g^−1^ and was comparable to the calcium concentration in sheep milk (202.83 mg 100 g^−1^). In comparison, the phosphorus content in these groups of ice cream ranged from 153.35 mg 100 g^−1^ to 157 mg 100 g^−1^. Adding inulin does not significantly increase the calcium and phosphorus content of ice cream. On the contrary, apple fiber contains 15.96 mg 100 g^−1^ of calcium and 51.80 mg 100 g^−1^ of phosphorus, resulting in a proportional increase in calcium and phosphorus in FLC (*L. casei*), FLA (*L. acidophilus*), FBB12 (*B. animalis*), FLP (*L. paracasei*) and FLR (*L. rahamnosus*) ice cream.

**Figure 2:**
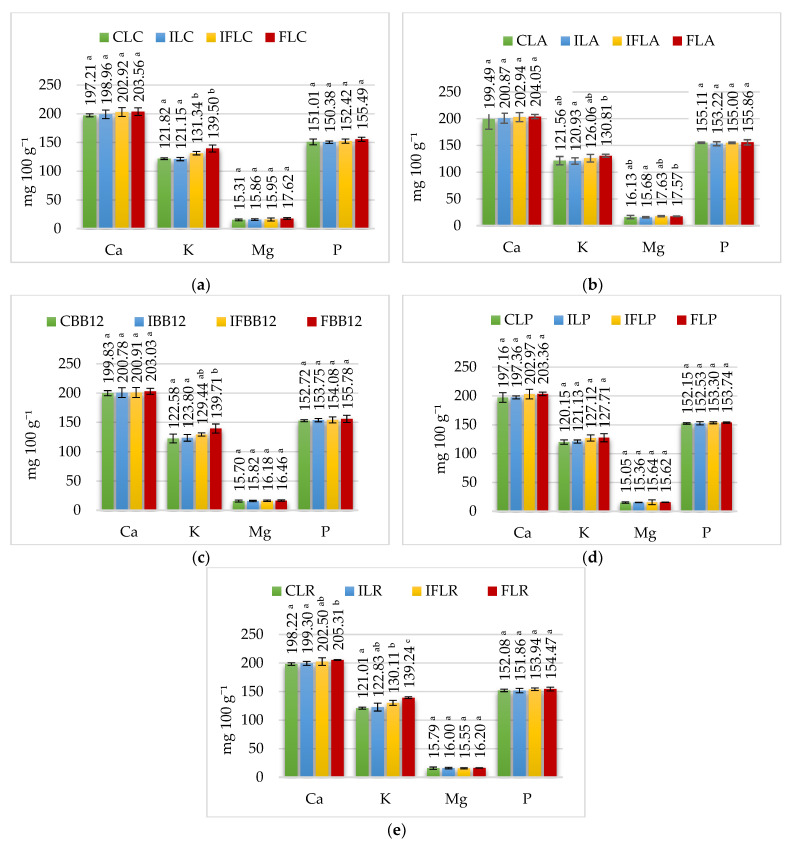
Macroelement content at the oral stage. (**a**) *L. casei*, (**b**) *L. acidophilus*, (**c**) *B. animalis*, (**d**) *L. paracasei*, (**e**) *L. rhamnosus. ^a–c^—*mean values denoted by different letters differ statistically significantly at *p* ≤ 0.05; CLC: control sample with *L. casei* 431; ILC: sample with 4% inulin and *L. casei* 431; IFLC: sample with 2.5% inulin and 1.5% apple fiber with *L. casei* 431; FLC: sample with 4% fiber and *L. casei* 431; CLA: control sample with *L. acidophilus*; ILA: sample with 4% inulin and *L. acidophilus*; IFLA: sample with 2.5% inulin and 1.5% apple fiber with *L. acidophilus*; FLA: sample with 4% fiber and *L. acidophilus*; CBB12: control sample with *B. animalis*; IBB12: sample with 4% inulin and *B. animalis*; IFBB12: sample with 2.5% inulin and 1.5% apple fiber with *B. animalis*; FBB12: sample with 4% fiber and *B. animalis*; CLP: control sample with *L. paracasei* L-26; ILP: sample with 4% inulin and *L. paracasei* L-26; IFLP: sample with 2.5% inulin and 1.5% apple fiber with *L. paracasei* L-26; FLP sample with 4% fiber and *L. paracasei* L-26; CLR: control sample with *L. rhamnosus*; ILR: sample with 4% inulin and *L. rhamnosus*; IFLR: sample with 2.5% inulin and 1.5% apple fiber with *L. rhamnosus*; FLR: sample with 4% fiber and *L. rhamnosus*.

In the mouth, ice cream samples are mixed with saliva and thus diluted, which contributes to a non-significant decrease in calcium concentration in all samples (Figure 2). On the contrary, the phosphorus content increased, which was caused by the presence of sodium hydrogen phosphate (Na_2_HPO_4_) in saliva. In humans, the amount of saliva secreted under resting conditions is 0.5 mL per minute, while after intense secretory stimulation with food, it can increase to 10 mL per minute [38].

When the ice cream enters the stomach during fasting, it comes into contact with a small amount (~50 mL) of gastric juice, which for an adult will have a pH between 1 and 2 [39]. However, considering the portion size of the ice cream (~100 mL) with a pH of 4.3–5.3, compared to the amount of gastric juice, the pH of the stomach will quickly rise to a value of about 4. Subsequently, the stomach ice cream is slightly diluted and acidified, resulting in the dissolution of some of the calcium phosphate present in the casein micelles [40,41]. Subsequently, gastric juice is secreted into the stomach, gradually lowering the pH [42].

**Figure 3:**
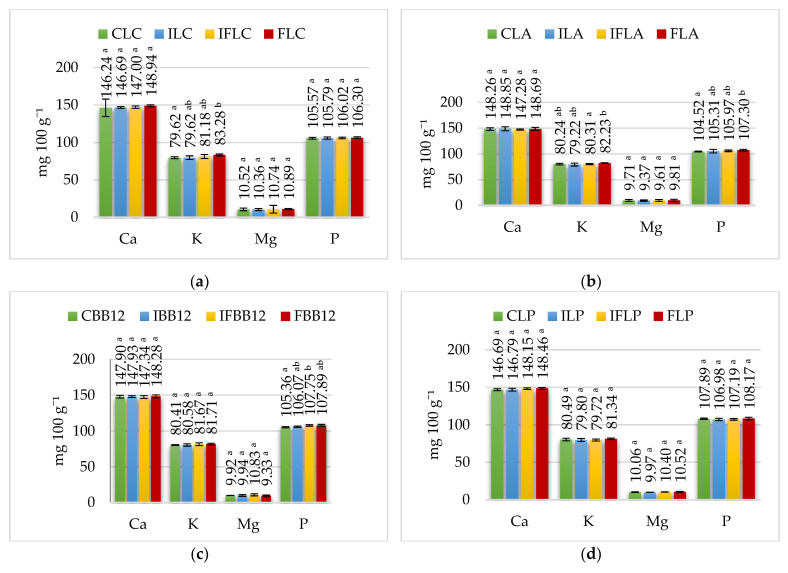

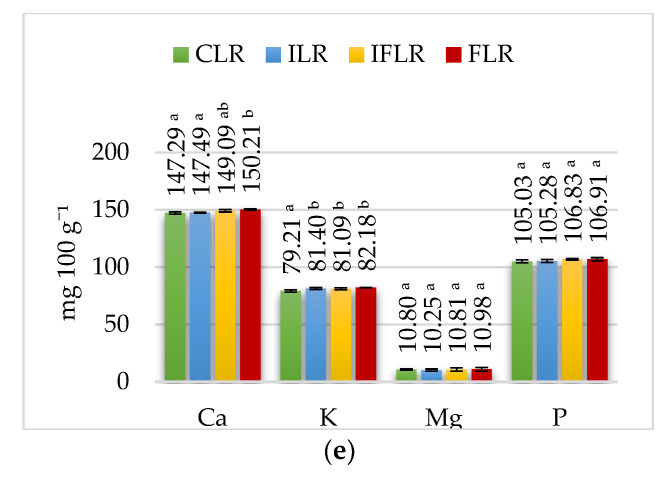
Macroelement content at the stomach stage. (**a**) *L. casei* (**b**) *L. acidophilus*, (**c**) *B. animalis*, (**d**) *L. paracasei*, (**e**) *L. rhamnosus. ^a^^–b^—*mean values denoted by different letters differ statistically significantly at *p* ≤ 0.05; CLC: control sample with *L. casei* 431; ILC: sample with 4% inulin and *L. casei* 431; IFLC: sample with 2.5% inulin and 1.5% apple fiber with *L. casei* 431; FLC: sample with 4% fiber and *L. casei* 431; CLA: control sample with *L. acidophilus*; ILA: sample with 4% inulin and *L. acidophilus*; IFLA: sample with 2.5% inulin and 1.5% apple fiber with *L. acidophilus*; FLA: sample with 4% fiber and *L. acidophilus*; CBB12: control sample with *B. animalis*; IBB12: sample with 4% inulin and *B. animalis*; IFBB12: sample with 2.5% inulin and 1.5% apple fiber with *B. animalis*; FBB12: sample with 4% fiber and *B. animalis*; CLP: control sample with *L. paracasei* L-26; ILP: sample with 4% inulin and *L. paracasei* L-26; IFLP: sample with 2.5% inulin and 1.5% apple fiber with *L. paracasei* L-26; FLP sample with 4% fiber and *L. paracasei* L-26; CLR: control sample with *L. rhamnosus*; ILR: sample with 4% inulin and *L. rhamnosus*; IFLR: sample with 2.5% inulin and 1.5% apple fiber with *L. rhamnosus*; FLR: sample with 4% fiber and *L. rhamnosus*.

Therefore, when the pH value is lower than 4 in the stomach, it should be sufficient to dissolve all calcium and phosphate from casein micelles [34]. Possible enzymatic coagulation of the micelles could impede this process and delay the release of calcium and phosphate from the ice cream. This delayed release may finally benefit ice cream’s calcium absorption due to the gastric phase’s reduced calcium concentration. However, dilution with gastric juice and slow release from the gastric coagulum would significantly reduce the concentration of calcium phosphate in the food content leaving the stomach [43]. In the gastric phase, the calcium concentration in the analyzed ice cream ranged from 146.24 mg 100 g^−1^ to 151.21 mg 100 g^−1^ (Figure 3), representing 72.98% to 73.32% of the calcium content of the ice cream before digestion. However, in the gastric phase, a significantly higher (*p* ≤ 0.05) calcium content was determined only in samples fermented by *L. rhamnosus*: the IFLR ice cream with inulin and fiber and FLR ice cream with fiber compared to control CLR ice cream and ILR ice cream with inulin. In other ice cream groups, added fiber had no effect on calcium content. In contrast, a significant effect of added apple fiber on the phosphorus content of the gastric phase was found in FLA (fermented by *L. acidophilus*) and FBB12 ice cream (fermented by *B. animalis*) compared to their control counterparts. Also notable was the tendency in the remaining ice cream with apple fiber to maintain higher phosphorus concentrations than those in samples without fiber.

**Figure 4:**
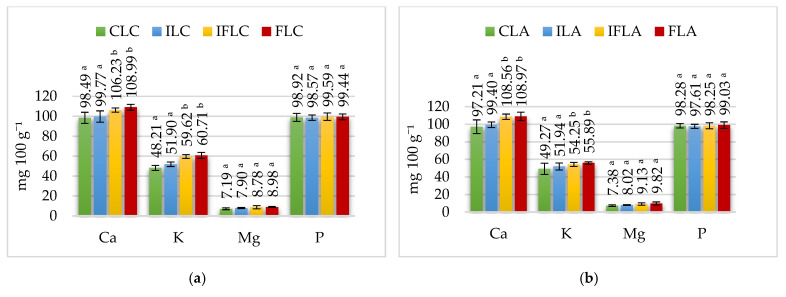

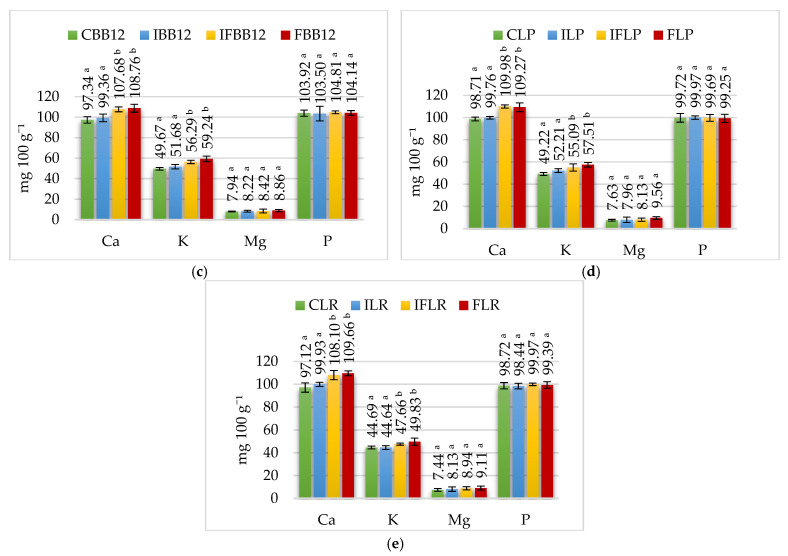
Macroelement content at the small intestine stage. (**a**) *L. casei* (**b**) *L. acidophilus*, (**c**) *B. animalis*, (**d**) *L. paracasei*, (**e**) *L. rhamnosus. ^a-b^ —*mean values denoted by different letters differ statistically significantly at *p* ≤ 0.05; CLC: control sample with *L. casei* 431; ILC: sample with 4% inulin and *L. casei* 431; IFLC: sample with 2.5% inulin and 1.5% apple fiber with *L. casei* 431; FLC: sample with 4% fiber and *L. casei* 431; CLA: control sample with *L. acidophilus*; ILA: sample with 4% inulin and *L. acidophilus*; IFLA: sample with 2.5% inulin and 1.5% apple fiber with *L. acidophilus*; FLA: sample with 4% fiber and *L. acidophilus*; CBB12: control sample with *B. animalis*; IBB12: sample with 4% inulin and *B. animalis*; IFBB12: sample with 2.5% inulin and 1.5% apple fiber with *B. animalis*; FBB12: sample with 4% fiber and *B. animalis*; CLP: control sample with *L. paracasei* L-26; ILP: sample with 4% inulin and *L. paracasei* L-26; IFLP: sample with 2.5% inulin and 1.5% apple fiber with *L. paracasei* L-26; FLP sample with 4% fiber and *L. paracasei* L-26; CLR: control sample with *L. rhamnosus*; ILR: sample with 4% inulin and *L. rhamnosus*; IFLR: sample with 2.5% inulin and 1.5% apple fiber with *L. rhamnosus*; FLR: sample with 4% fiber and *L. rhamnosus*.

The rapidity of gastric emptying depends on many factors, including volume flow restrictions, caloric density, pH and rheological properties [43]. Phosphorus absorption occurs in the duodenum (35%), jejunum (25%) and ileum (40%) [44]. The naturally occurring phosphorus in food is absorbed from the gastrointestinal tract at 40–60% [37]. The phosphorus content in the tested samples in the intestinal phase is shown in Figure 4. The results of the bioavailability of macronutrients are shown in Table 7, Table 8, Table 9, Table 10 and Table 11.

The bioavailability of phosphorus in the ice cream ranged from 47.82% in FLC ice cream with fiber fermented by *L. casei* to 50.94% in the FBB12 group with fiber addition fermented by *B. animalis*. Adding apple fiber did not significantly reduce the bioavailability of phosphorus in ice cream fermented by *L. casei*, *L. paracasei* and *L. rhamnosus*, nor did it significantly increase bioavailability in ice cream with *L. acidophilus* (FLA) and B. animalis (FBB12), compared to control counterparts. The highest bioavailability of phosphorus (>50%) was found in sheep’s milk ice cream fermented by *B. animalis*. The remaining sheep’s milk ice creams had lower phosphorus bioavailability by 1–2.5%.

The bioavailability of calcium in the tested ice cream ranged from 40.63% in FBB12 (with fiber, fermented by *B. animalis*) to 54.40% in control CLP samples fermented by *L. paracasei* (Table 7, Table 8, Table 9, Table 10 and Table 11). The highest bioavailability of calcium, depending on the type of bacteria used in each group, was always shown for the control samples. However, adding 4% inulin already reduced calcium bioavailability by about 3–5%, and a mixture of 2.5% inulin with 1.5% apple fiber reduced calcium bioavailability by 4–8% compared to controls. Adding 4% apple fiber significantly reduced the bioavailability of calcium from sheep’s milk ice cream by up to 6–12%. FLR ice cream with fiber fermented by *L. rhamnosus* showed a significant reduction in the bioavailability of >12% compared to control CLR ice cream. Our study and the two-factor analysis of variance (ANOVA) show that the bioavailability of calcium from sheep’s milk ice cream is significantly influenced by the two factors analyzed, which are the fiber (*p* = 0.0000) and the type of bacteria (*p* = 0.0001), as well as interactions between these factors (*p* = 0.0006). Also, a study by Bosscher et al. [45] showed that dietary fiber inhibits mineral bioavailability more in casein than in whey-based formulations.

Most calcium (about 65%) is absorbed at pH 6.5–7.5. It should be noted that calcium is not absorbed from the stomach [46]. In order to cross the intestinal barrier, calcium must be in soluble form, generally ionized (Ca^2+^) or bound to a soluble organic molecule. The auxiliary organs supporting intestinal digestion and absorption are the pancreas, liver and gallbladder [38]. Calcium transport involves both active and passive transport mechanisms. Active transport occurs mainly in the duodenum and upper jejunum [47]. In the ileum, the primary absorption mechanism is passive, since food moves slowly through this area of the gastrointestinal tract. The small intestine is responsible for more than 90% of total calcium uptake in humans, while about 3–6% of calcium is absorbed in the large intestine, depending on calcium loading [48,49]. A study by Szalast-Pietrzak et al. [50] on food products showed the highest percentage of calcium bioavailability from natural yogurt, at 37.73%.

Our study of the effect of applied probiotic strains on calcium bioavailability also supports the results of Sharifi-Rad et al. [51], where one possible mechanism by which calcium availability is increased is higher calcium absorption and fermentation in the intestine by probiotics. Gilman and Cashman [52] previously reported that in human intestinal Caco-2 cells in culture, *Lactobacillus salivarius* could increase Ca^2+^ uptake, although exposure of Caco-2 cells to probiotics has no effect on Ca^2+^ transport.

### 3.2. Magnesium

Sheep milk provides 16–18 mg 100 g^−1^ of magnesium, which occurs in the form of soluble compounds (about 73–75% of total Mg) and colloidal compounds (phosphates, citrates). Only about 15% of magnesium is presented in an ionized form. [3]. The results determining the magnesium content in the analyzed ice cream groups are shown in Table 2, Table 3, Table 4, Table 5 and Table 6. In the control ice cream, the content of this macroelement was determined in the range from 15.96 mg 100 g^−1^ in control ice cream with *L. casei* (CLC) to 17.52 mg 100 g^−1^ in control ice cream with *L. paracasei* (CLP). Adding apple fiber significantly increased the magnesium content of FLC (*L. casei*), FLA (*L. acidophilus*), FBB12 (*B. animalis*), FLP (*L. paracasei*) and FLR (*L. rhamnosus*) ice cream by about 0.5–1.4 mg 100 g^−1^ due to the presence of magnesium in apple fiber (Table 1). In contrast, the addition of inulin did not change the magnesium content in the ice cream before digestion compared to its control counterparts. The concentration of magnesium in ice cream in the oral, stomach and intestine stages, depending on the addition of fiber and probiotic strain, is shown in Figure 2, Figure 3 and Figure 4.

The bioavailability of magnesium from sheep’s milk products is poorly studied. In our study, the bioavailability of magnesium in sheep’s milk ice cream ranged from 55.64% in the control ice cream fermented by *L. casei* (CLC) to 44.42% in the group with fiber fermented by *L. acidophilus* (FLA). The bioavailability of magnesium was highest in the control samples, where it always exceeded 50%. The highest bioavailability was determined in the control CLC ice cream fermented by *L. casei*. The addition of fiber had an essential role in modifying the bioavailability of magnesium. Hussain et al. [53] indicated that apple fiber contains 40% cellulose and 19% water-soluble hemicellulose. Also, as a polysaccharide, inulin is a low-molecular-weight polymer with poor water solubility, contributing to faster intestinal transit. However, it should be mentioned that adding inulin only reduced magnesium’s bioavailability by about 5–6% compared to control counterparts. In contrast, adding apple fiber reduced the bioavailability of magnesium in the ice cream by 8.03% in FLA (fermented by *L. acidophilus*), 7.48% in FLC (fermented by *L. casei*) and 7.25% in the FLR (fermented by *L. rhamnosus*) group. Ice cream with fiber addition, FBB12 fermented by *B. animalis* and FLP fermented by *L. paracasei*, showed 5–6% lower magnesium bioavailability than controls.

Bielik et al. [22] consider that the gut microflora influences mineral metabolism by directly influencing mineral absorption in the digestive tract during digestion and producing several enzymes that help release minerals from food. These include bacterial phytases, which catalyze the hydrolysis of phytic acid found in many plant tissues, releasing proper forms of minerals such as calcium, magnesium and phosphorus [54,55]. Aljevich et al. [56] observed a higher bioavailability of magnesium and other minerals from cheese when combined with probiotics. Cultures of *Lactobacillus* spp. consumed with Dutch-type cheese increased Mg (~18%) and Ca (~2.5%) availability in vitro [28]. Similarly, fermented goat milk containing Lactobacillus plantarum increased Mg bioavailability compared to commercial fermented goat milk [21].

This implies that the bacterial strain conducting the fermentations of the ice cream mixes also significantly affects magnesium bioavailability (*p* = 0.0001), as confirmed by a two-factor analysis of variance (ANOVA). The calculations show that the bioavailability of magnesium from sheep’s milk ice cream is significantly influenced by both factors analyzed, including the fiber (*p* = 0.0011) and the interactions between these factors (*p* = 0.0005).

Comparable values for magnesium absorption from various diets to those in our study have been published [49]. In an equilibrium study conducted on healthy young men, the apparent magnesium absorption from a mixed Western diet containing 18 g of fiber daily was 46.3%. Knudsen et al. [57] found an average magnesium absorption of 46%. In another study, magnesium absorption from milk was measured in adolescents aged 9–14 years using the stable isotope multiscale technique. The absorption of magnesium from milk was 42.8% among girls and 45.3% among boys and did not differ significantly between the sexes [58].

### 3.3. Potassium

Due to the high potassium content in apple fiber (Table 1), its addition to ice cream mixes significantly increased the amount of potassium in sheep’s milk ice cream (Table 2, Table 3, Table 4, Table 5 and Table 6). The concentration of potassium in ice cream in the mouth, stomach and intestine depending on the addition of fiber and the probiotic strain used for fermentation is shown in Figure 2, Figure 3 and Figure 4.

Potassium is intrinsically soluble and rapidly diffuses into the lumen of the upper gastrointestinal tract. The small intestine is the primary location for potassium absorption, with approximately 90% of dietary potassium being absorbed by passive diffusion [59,60]. However, relatively little is known about the bioavailability of potassium, and most work has focused on assessing urinary potassium loss after potassium salt supplementation [61,62,63]. Only potato has been studied for potassium bioavailability, and this food consists mainly of easily digestible starch [59].

In our study, the bioavailability of potassium was higher in control ice cream than in ice cream with fiber (Table 7, Table 8, Table 9, Table 10 and Table 11). In the control ice cream (CLC, CLA, CBB12, CLP, CLR), the bioavailability of potassium was about 60%. In ice cream with inulin, it was lower by 3–4%, and in ice cream with apple fiber, it was lower by 6–9%. A two-factor analysis of variance (ANOVA) shows that the bioavailability of potassium in sheep’s milk ice cream is significantly affected by the addition of fiber (*p* = 0.0000), while the type of bacteria (*p* = 0.6731) and interactions between these factors (*p* = 0.7456) were not significant.

The bioavailability of potassium in whole fruits and vegetables can be as high as 50% to 60%, and there is a lack of evidence linking higher consumption of fruits and vegetables to higher serum potassium concentrations [64]. The bioavailability of potassium from food additives can be as high as 100% [64,65]. Picard [65] and MacDonald-Clarke et al. [66] also found that the bioavailability of potassium from fruits and vegetables is 50–60%, compared to 90% from animal protein and 95% from additives. Only a few studies show how well the various forms of potassium contained in dietary supplements are absorbed. A dose–response study showed that people absorb about 94% of the potassium gluconate in supplements, and the absorption rate is similar to that of potassium from potatoes [66].

## 4. Conclusions

In general, considering macronutrients in food matrices and their changes during digestion is crucial for allowing the optimal use of macroelements in the diet. The results of the study confirmed the beneficial effect of the bacterial strain on the bioavailability of Ca, Mg and P and, thus, their support for the human gut microbiome. Further research should address the action of specific bacterial species and their effect on improving or preventing micronutrient deficiencies. Similarly, a better understanding of the underlying mechanisms by which the gut microbiota influences host micronutrient uptake and absorption would enable a postbiotic approach to match micronutrient availability with host needs.

## Figures and Tables

**Table 1:** Macroelement content (mg 100 g^−1^) in ice cream with L. casei.

Macroelement	CLC	ILC	IFLC	FLC
Ca	200.36 ^a^ ± 2.48	203.63 ^a^ ± 0.63	205.70 ^b^ ± 1.86	208.90 ^b^ ± 2.76
K	124.78 ^a^ ± 1.66	124.89 ^a^ ± 1.32	135.59 ^b^ ± 1.79	141.57 ^c^ ± 1.20
Mg	15.96 ^a^ ± 0.25	16.02 ^a^ ± 0.21	17.07 ^b^ ± 0.28	17.40 ^b^ ± 0.24
P	154.35 ^a^ ± 0.22	154.08 ^a^ ± 0.67	158.36 ^b^ ± 0.20	159.37 ^c^ ± 1.96
Ca:P	1.29:1	1.32:1	1.29:1	1.31:1

^a–c^—mean values denoted in rows by different letters differ statistically significantly at *p* ≤ 0.05; CLC: control sample with *L. casei* 431; ILC: sample with 4% inulin and *L. casei* 431; IFLC: sample with 2.5% inulin and 1.5% apple fiber with *L. casei* 431; FLC: sample with 4% fiber and *L. casei* 431.

**Table 2:** Macroelement content (mg 100 g^−1^) in ice cream with *L. acidophilus*.

Macroelement	CLA	ILA	IFLA	FLA
Ca	202.45 ^a^ ± 1.35	203.57 ^ab^ ± 1.33	204.25 ^b^ ± 1.63	208.20 ^c^ ± 2.51
K	125.34 ^a^ ± 1.31	124.96 ^a^ ± 0.44	129.59 ^b^ ± 0.65	139.18 ^c^ ± 0.80
Mg	16.55 ^a^ ± 0.33	17.43 ^a^ ± 0.12	17.94 ^b^ ± 0.15	18.01 ^b^ ± 0.24
P	157.00 ^a^ ± 0.92	157.83 ^a^ ± 0.50	159.13 ^b^ ± 0.45	160.67 ^c^ ± 0.18
Ca:P	1.28:1	1.28:1	1.28:1	1.29:1

^a–c^—mean values denoted in rows by different letters differ statistically significantly at *p* ≤ 0.05; CLA: control sample with *L. acidophilus*; ILA: sample with 4% inulin and *L. acidophilus*; IFLA: sample with 2.5% inulin and 1.5% apple fiber with *L. acidophilus*; FLA: sample with 4% fiber and *L. acidophilus*.

**Table 3:** Macroelement content (mg 100 g^−1^) in ice cream with *B. animalis*.

Macroelement	CBB12	IBB12	IFBB12	FBB12
Ca	201.97 ^a^ ± 1.22	202.63 ^a^ ± 0.76	203.85 ^b^ ± 0.87	206.59 ^c^ ± 0.37
K	125.55 ^a^ ± 1.94	124.81 ^a^ ± 0.64	131.27 ^b^ ± 1.77	141.10 ^c^ ± 0.84
Mg	16.53 ^a^ ± 0.36	16.60 ^a^ ± 0.10	16.83 ^a^ ± 0.35	17.34 ^b^ ± 0.19
P	156.50 ^a^ ± 0.68	157.42 ^a^ ± 0.45	158.50 ^b^ ± 0.32	159.92 ^c^ ± 0.13
Ca:P	1.29:1	1.28:1	1.28:1	1.29:1

^a–c^—mean values denoted in rows by different letters differ statistically significantly at *p* ≤ 0.05; CBB12: control sample with *B*. *animalis*; IBB12: sample with 4% inulin and *B*. *animalis*; IFBB12: sample with 2.5% inulin and 1.5% apple fiber with *B*. *animalis*; FBB12: sample with 4% fiber and *B*. *animalis*.

**Table 4:** Macroelement content (mg 100 g^−1^) in ice cream with *L. paracasei*.

Macroelement	CLP	ILP	IFLP	FLP
Ca	199.47 ^a^ ± 1.09	201.82 ^a^ ± 0.73	203.90 ^b^ ± 0.57	205.40 ^c^ ± 0.78
K	125.37 ^a^ ± 0.99	125.68 ^a^ ± 1.04	130.28 ^b^ ± 0.55	142.02 ^c^ ± 0.90
Mg	17.52 ^a^ ± 0.16	17.64 ^a^ ± 0.08	17.88 ^b^ ± 0.08	18.02 ^b^ ± 0.21
P	154.03 ^a^ ± 0.98	154.90 ^a^ ± 0.72	157.45 ^b^ ± 0.34	159.46 ^c^ ± 1.05
Ca:P	1.29:1	1.30:1	1.29:1	1.28:1

^a–c^—mean values denoted in rows by different letters differ statistically significantly at *p* ≤ 0.05; CLP: control sample with *L. paracasei* L-26; ILP: sample with 4% inulin and *L. paracasei* L-26; IFLP: sample with 2.5% inulin and 1.5% apple fiber with *L. paracasei* L-26; FLP sample with 4% fiber and *L. paracasei* L-26.

**Table 5:** Macroelement content (mg 100 g^−1^) in ice cream with *L. rhamnosus*.

Macroelement	CLR	ILR	IFLR	FLR
Ca	200.83 ^a^ ± 1.31	201.38 ^a^ ± 0.60	203.13 ^b^ ± 0.85	206.23 ^c^ ± 1.49
K	124.83 ^a^ ± 0.68	125.17 ^a^ ± 0.92	133.19 ^b^ ± 0.53	142.23 ^c^ ± 1.51
Mg	16.34 ^a^ ± 0.12	16.67 ^a^ ± 0.21	16.41 ^a^ ± 0.13	17.44 ^b^ ± 0.37
P	154.93 ^a^ ± 1.10	155.04 ^a^ ± 0.93	158.56 ^b^ ± 0.54	160.17 ^b^ ± 0.57
Ca:P	1.29:1	1.29:1	1.28:1	1.28:1

^a–c^—mean values denoted in rows by different letters differ statistically significantly at *p* ≤ 0.05; CLR: control sample with *L. rhamnosus*; ILR: sample with 4% inulin and *L. rhamnosus*; IFLR: sample with 2.5% inulin and 1.5% apple fiber with *L. rhamnosus*; FLR: sample with 4% fiber and *L. rhamnosus*.

**Table 6:** Macroelement content (mg 100 g^−1^) in raw materials.

Macroelement	Inulin	Apple Fiber	Sheep Milk
Ca	1.42 ^a^ ± 0.12	15.96 ^b^ ± 0.21	202.83 ^c^ ± 2.53
K	4.73 ^a^ ± 0.69	398.93 ^c^ ± 0.53	146.22 ^b^ ± 1.57
Mg	0.05 ^a^ ± 0.00	15.11 ^c^ ± 0.39	15.96 ^b^ ± 2.01
P	0.20 ^a^ ± 0.03	51.80 ^c^ ± 0.01	156.23 ^b^ ± 2.53

^a–c^—mean values denoted in rows by different letters differ statistically significantly at *p* ≤ 0.05.

**Table 7:** Bioavailability of macroelements (%) in ice cream with *L. casei*.

Macroelement	CLC	ILC	IFLC	FLC
Ca	48.81 ^d^ ± 1.53	46.66 ^c^ ± 1.66	43.39 ^b^ ± 1.14	42.41 ^a^ ± 1.96
K	60.66 ^d^ ± 2.54	57.01 ^c^ ± 2.28	53.85 ^b^ ± 1.87	51.42 ^a^ ± 3.01
Mg	55.64 ^b^ ± 0.96	49.93 ^a^ ± 0.54	48.68 ^a^ ± 1.64	48.16 ^a^ ± 0.47
P	49.26 ^a^ ± 4.04	49.05 ^a^ ± 2.72	48.74 ^a^ ± 3.70	47.82 ^a^ ± 2.96

^a–d^—mean values denoted in rows by different letters differ statistically significantly at *p* ≤ 0.05; CLC: control sample with *L. casei* 431; ILC: sample with 4% inulin and *L. casei* 431; IFLC: sample with 2.5% inulin and 1.5% apple fiber with *L. casei* 431; FLC: sample with 4% fiber and *L. casei* 431.

**Table 8:** Bioavailability of macroelements (%) in ice cream with *L. acidophilus*.

Macroelement	CLA	ILA	IFLA	FLA
Ca	51.70 ^c^ ± 3.23	47.09 ^b^ ± 2.61	43.84 ^a^ ± 2.01	40.77 ^a^ ± 1.85
K	60.85 ^c^ ± 0.69	57.06 ^b^ ± 1.02	54.80 ^a^ ± 1.11	54.45 ^a^ ± 0.89
Mg	52.45 ^c^ ± 2.25	47.12 ^b^ ± 1.58	46.88 ^b^ ± 1.51	44.42 ^a^ ± 2.01
P	49.19 ^a^ ± 0.52	49.36 ^a^ ± 0.34	49.36 ^a^ ± 0.56	49.48 ^a^ ± 0.47

^a–c^—mean values denoted in rows by different letters differ statistically significantly at *p* ≤ 0.05; CLA: control sample with *L. acidophilus*; ILA: sample with 4% inulin and *L. acidophilus*; IFLA: sample with 2.5% inulin and 1.5% apple fiber with *L. acidophilus*; FLA: sample with 4% fiber and *L. acidophilus*.

**Table 9:** Bioavailability of macroelements (%) in ice cream with *B. animalis*.

Macroelement	CBB12	IBB12	IFBB12	FBB12
Ca	52.21 ^c^ ± 1.32	47.56 ^b^ ± 1.03	46.06 ^b^ ± 0.98	40.63 ^a^ ± 0.96
K	60.06 ^c^ ± 1.06	56.28 ^b^ ± 0.65	56.00 ^b^ ± 0.59	51.34 ^a^ ± 1.02
Mg	52.15 ^b^ ± 1.12	46.08 ^a^ ± 1.09	46.46 ^a^ ± 1.03	46.02 ^a^ ± 1.11
P	50.54 ^a^ ± 0.54	50.51 ^a^ ± 0.55	50.46 ^a^ ± 0.63	50.94 ^a^ ± 0.87

^a–c^—mean values denoted in rows by different letters differ statistically significantly at *p* ≤ 0.05; CBB12: control sample with *B*. *animalis*; IBB12: sample with 4% inulin and *B*. *animalis*; IFBB12: sample with 2.5% inulin and 1.5% apple fiber with *B*. *animalis*; FBB12: sample with 4% fiber and *B*. *animalis*.

**Table 10:** Bioavailability of macroelements (%) in ice cream with *L. paracasei*.

Macroelement	CLP	ILP	IFLP	FLP
Ca	54.40 ^d^ ± 1.21	48.50 ^c^ ± 0.95	45.84 ^b^ ± 1.02	41.92 ^a^ ± 1.32
K	60.25 ^d^ ± 1.06	56.28 ^c^ ± 0.98	53.82 ^b^ ± 0.65	50.55 ^a^ ± 0.78
Mg	51.32 ^b^ ± 0.95	46.96 ^a^ ± 0.56	46.21 ^a^ ± 0.21	46.07 ^a^ ± 0.47
P	49.29 ^a^ ± 0.72	49.19 ^a^ ± 0.54	49.15 ^a^ ± 0.36	48.91 ^a^ ± 0.72

^a–d^—mean values denoted in rows by different letters differ statistically significantly at *p* ≤ 0.05; CLP: control sample with *L. paracasei* L-26; ILP: sample with 4% inulin and *L. paracasei* L-26; IFLP: sample with 2.5% inulin and 1.5% apple fiber with *L. paracasei* L-26; FLP sample with 4% fiber and *L. paracasei* L-26.

**Table 11:** Bioavailability of macroelements (%) in ice cream with *L. rhamnosus*.

Macroelement	CLR	ILR	IFLR	FLR
Ca	49.91 ^d^ ± 1.15	46.98 ^c^ ± 1.24	45.03 ^b^ ± 0.67	43.05 ^a^ ± 1.96
K	60.51 ^d^ ± 2.02	56.34 ^c^ ± 1.08	53.06 ^b^ ± 1.09	50.82 ^a^ ± 1.12
Mg	53.18 ^c^ ± 0.79	48.05 ^b^ ± 0.98	48.02 ^b^ ± 0.57	45.93 ^a^ ± 0.24
P	49.18 ^a^ ± 2.05	49.21 ^a^ ± 1.01	49.10 ^a^ ± 2.04	48.92 ^a^ ± 1.59

^a–d^—mean values denoted in rows by different letters differ statistically significantly at *p* ≤ 0.05; CLR: control sample with *L. rhamnosus*; ILR: sample with 4% inulin and *L. rhamnosus*; IFLR: sample with 2.5% inulin and 1.5% apple fiber with *L. rhamnosus*; FLR: sample with 4% fiber and *L. rhamnosus*.

## Data Availability

The original data presented in the study are included in the article; further inquiries can be directed to the corresponding author.
